# Supplementary material for: Moesin Serves as Scaffold Protein for PD-L1 in Human Uterine Cervical Squamous Carcinoma Cells
Source: J Clin Med. 2022 Jul 1;11(13):3830. doi: 10.3390/jcm11133830 (PMC9267616; doi:10.3390/jcm11133830)
Supplement: Supplementary file 1 [file jcm-11-03830-s001.zip › jcm-1739989-supplementary.pdf]

Supplementary Materials

Moesin Serves as Scaffold Protein for PD–L1 in Human Uterine Cervical Squamous Carcinoma Cells

Rina Doukuni [1], Takuro Kobori [1,*], Chihiro Tanaka [1], Mayuka Tameishi [1], Yoko Urashima [1], Takuya Ito [2] and Tokio Obata [1,*]

[1] Laboratory of Clinical Pharmaceutics, Faculty of Pharmacy, Osaka Ohtani University, Tondabayashi, 584-8540 Osaka, Japan; u4118098@osaka-ohtani.ac.jp (R.D.); u4117078@osaka-ohtani.ac.jp (C.T.); u4117083@osaka-ohtani.ac.jp (M.T.); urasiyo@osaka-ohtani.ac.jp (Y.U.)
[2] Laboratory of Natural Medicines, Faculty of Pharmacy, Osaka Ohtani University, Tondabayashi, 584-8540 Osaka, Japan; itoutaku@osaka-ohtani.ac.jp (T.I.)
* Correspondence: koboritaku@osaka-ohtani.ac.jp (T.K.); obatatoki@osaka-ohtani.ac.jp (T.O.); Tel.: +81-721-24-9374 (T.K.); +81-721-24-9371 (T.O.)

Negative fluorescence staining of no-primary-antibody control BOKU cells and HCS-2 Cells in confocal laser scanning microscopy

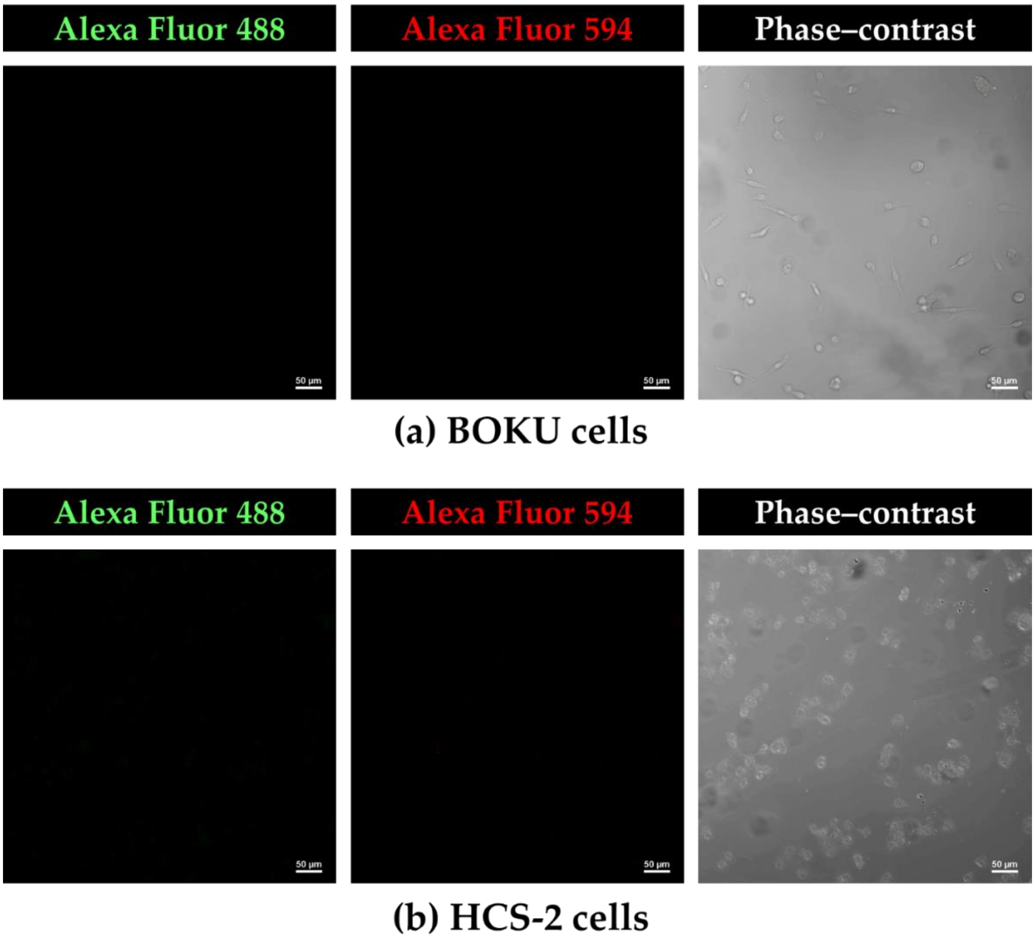

Figure S1. Negative fluorescence staining of no-primary-antibody control BOKU cells and HCS-2 cells in confocal laser scanning microscopy. **(a)** BOKU cells, **(b)** HCS-2 cells**.** Left and middle; Fluorescence images of goat anti-rabbit IgG (H+L) secondary antibodies conjugated with an Alexa Fluor 488 or an Alexa Fluor 594, respectively, without primary antibodies. Right; Phase-contrast image. Scale bars: 50 µm. All images were captured by confocal laser scanning microscopy.

Original immunoblot images

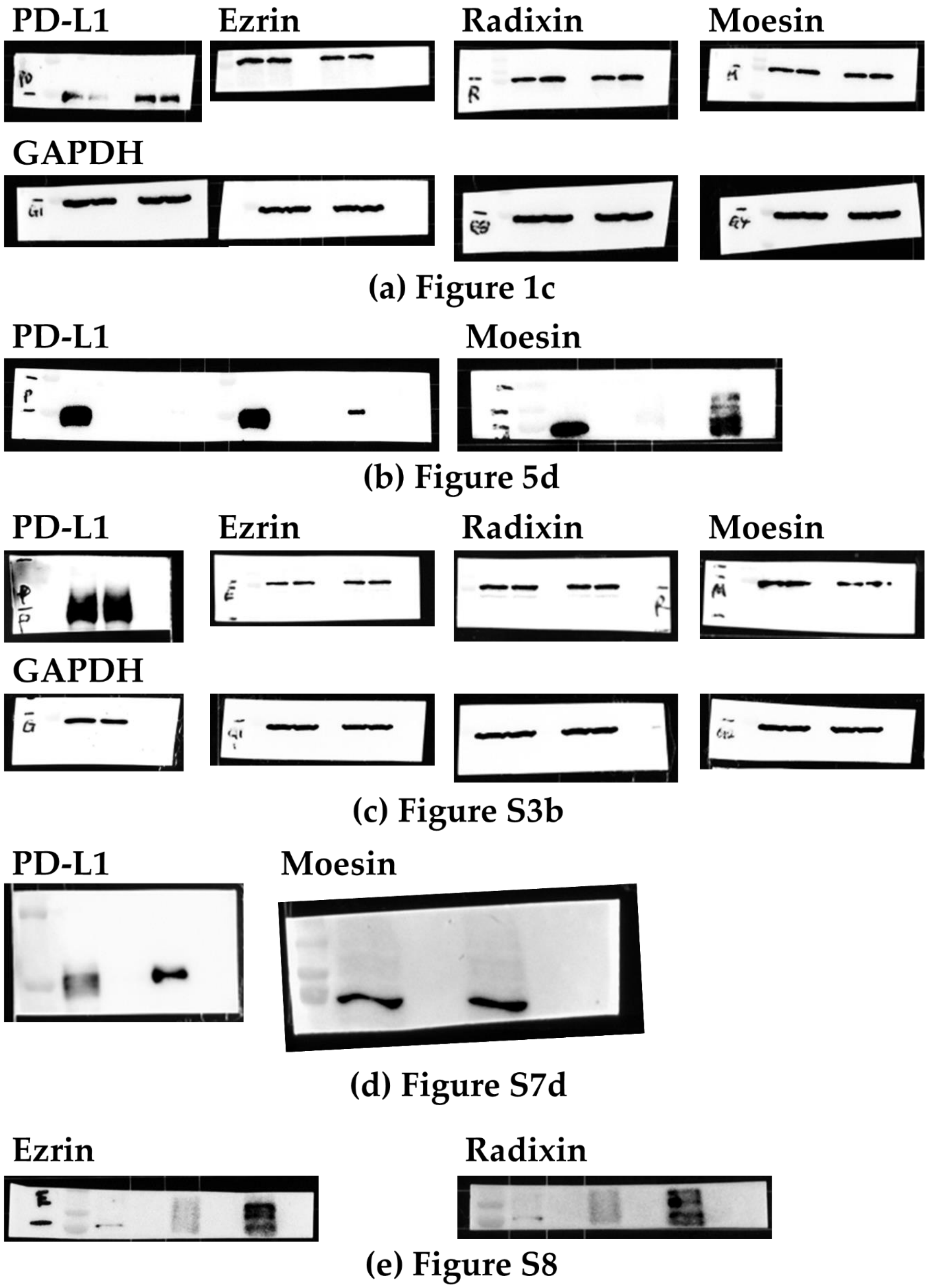

Figure S2. Original immunoblot images. The original western blotting membrane to detect the protein expression of programmed death ligand-1 (PD-L1), ezrin, radixin, and moesin as well as the corresponding glyceraldehyde-3-phosphate dehydrogenase (GAPDH) used as a loading control shown in (**a**) Figure 1c, (**b**) Figure 5d, (**c**) Figure S3b, (**d**) Figure S7d, and (**e**) Figure S8.

Gene and protein expression profiles of programmed death ligand-1 (PD-L1), ezrin, radixin, and moesin (ERM) in HCS-2 cells

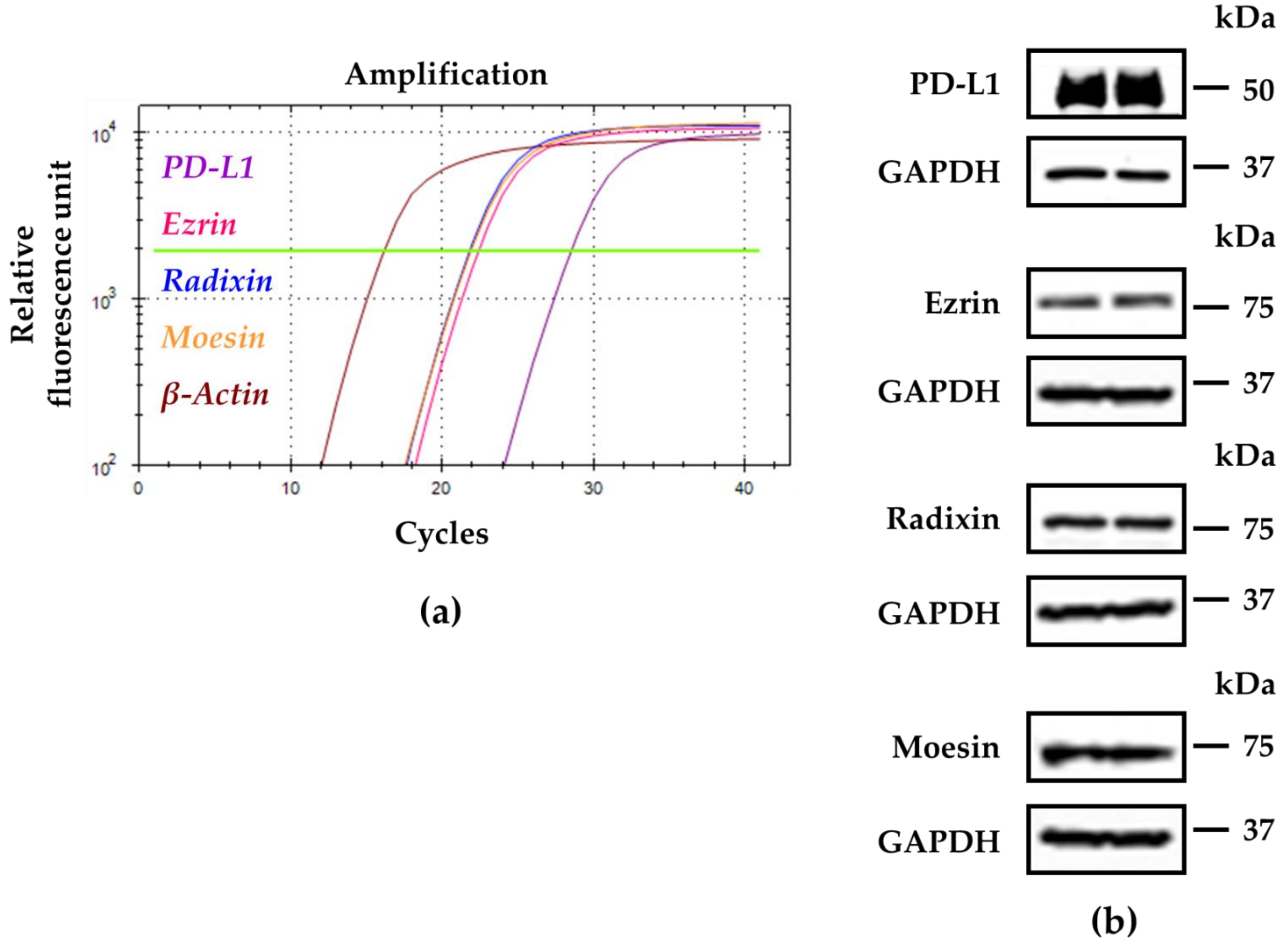

Figure S3. Gene and protein expression profiles of programmed death ligand-1 (PD-L1), ezrin, radixin, and moesin (ERM) in HCS-2 cells. (**a**) Representative amplification curves for PD-L1 and the ERM proteins as well as β-actin, included as an internal control, in HCS-2 cells as determined by real-time reverse transcription polymerase chain reaction (RT-PCR). (**b**) Representative immunoblot images for PD-L1 and the ERM proteins as well as glyceraldehyde-3-phosphate dehydrogenase (GAPDH), included as an internal control, in whole-cell lysates of HCS-2 cells. Molecular weights are denoted in kDa. Data are representative of three independent experiments using at least three independent samples of total RNA and protein extracts.

Confocal laser scanning microscopy (CLSM) analysis of the intracellular distribution of PD-L1 and the ERM family in HCS-2 cells

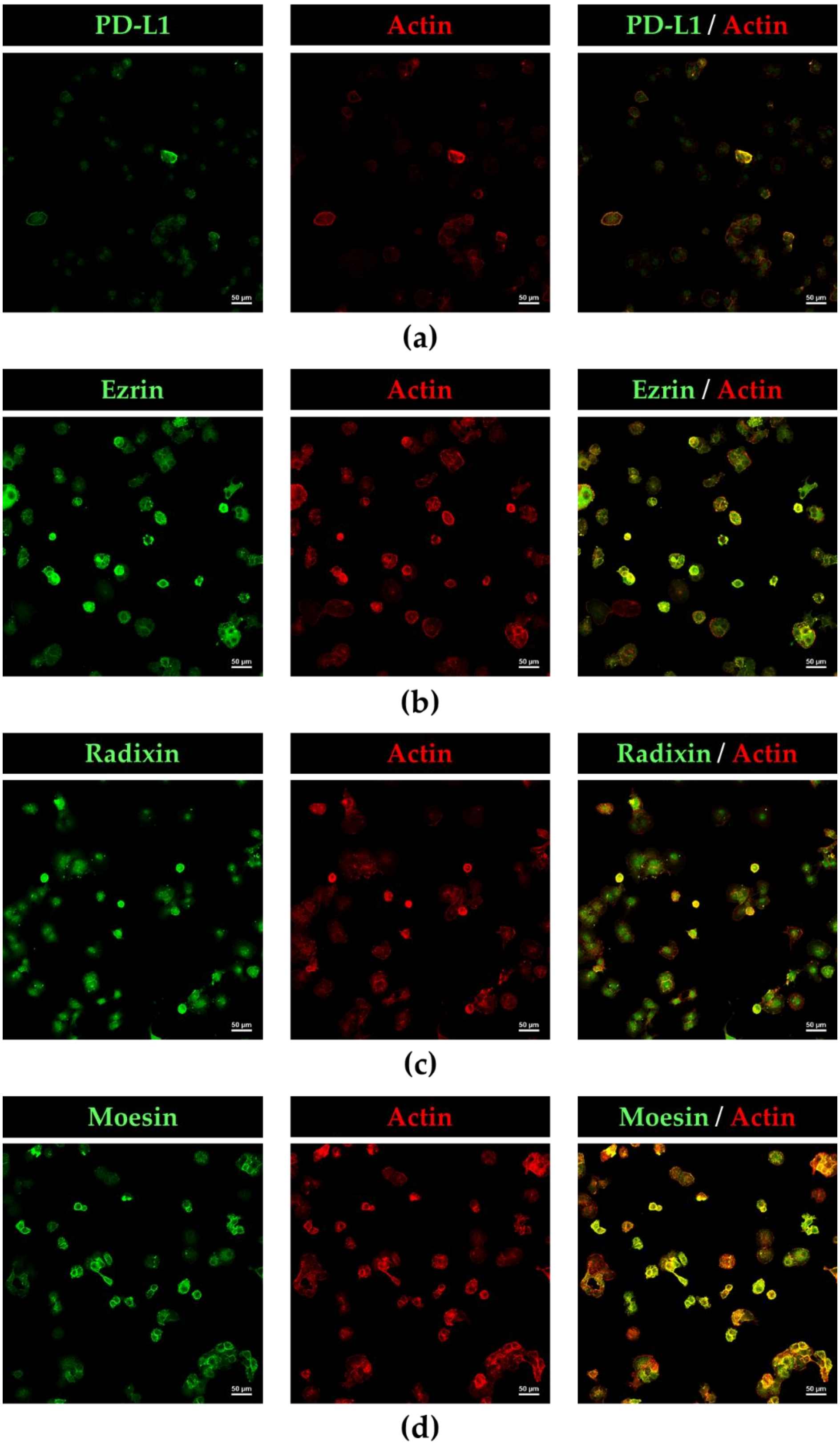

Figure S4. Confocal laser scanning microscopy (CLSM) analysis of the intracellular distribution of PD-L1 and the ERM family in HCS-2 cells. (**a**) PD-L 1, (**b**) ezrin, (**c**) radixin, and (**d**) moesin labeled with Alexa Fluor 488 (green) physically colocalized with F-actin labeled with tetramethylrhodamine (red). Scale bars: 50 μm. All images are representative of at least three independent experiments.

Colocalization of PD-L1 with the ERM family in the plasma membrane of HCS-2 cells

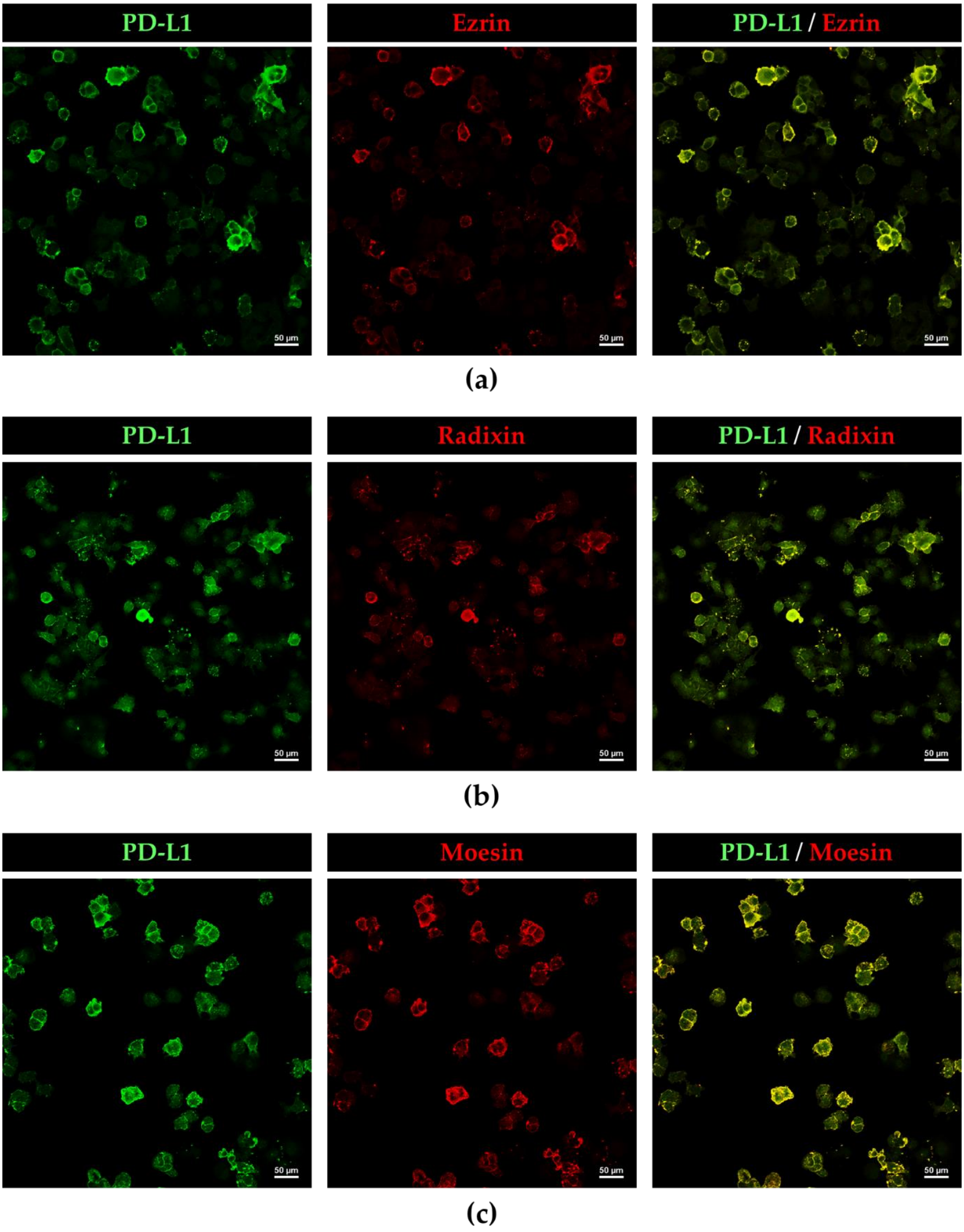

Figure S5. Colocalization of PD-L1 with the ERM family in the plasma membrane of HCS-2 cells. CLSM analysis showed that PD-L1 labeled with Alexa Fluor 488 (green) strongly colocalized with (**a**) ezrin, (**b**) radixin, and (**c**) moesin labeled with Alexa Fluor 594 (red) in the plasma membrane. Scale bar 50 µm. All images are representative of at least three independent experiments.

Effects of RNA interference-mediated knockdown of the ERM family on target mRNA levels in and cell viability of HCS-2 cells

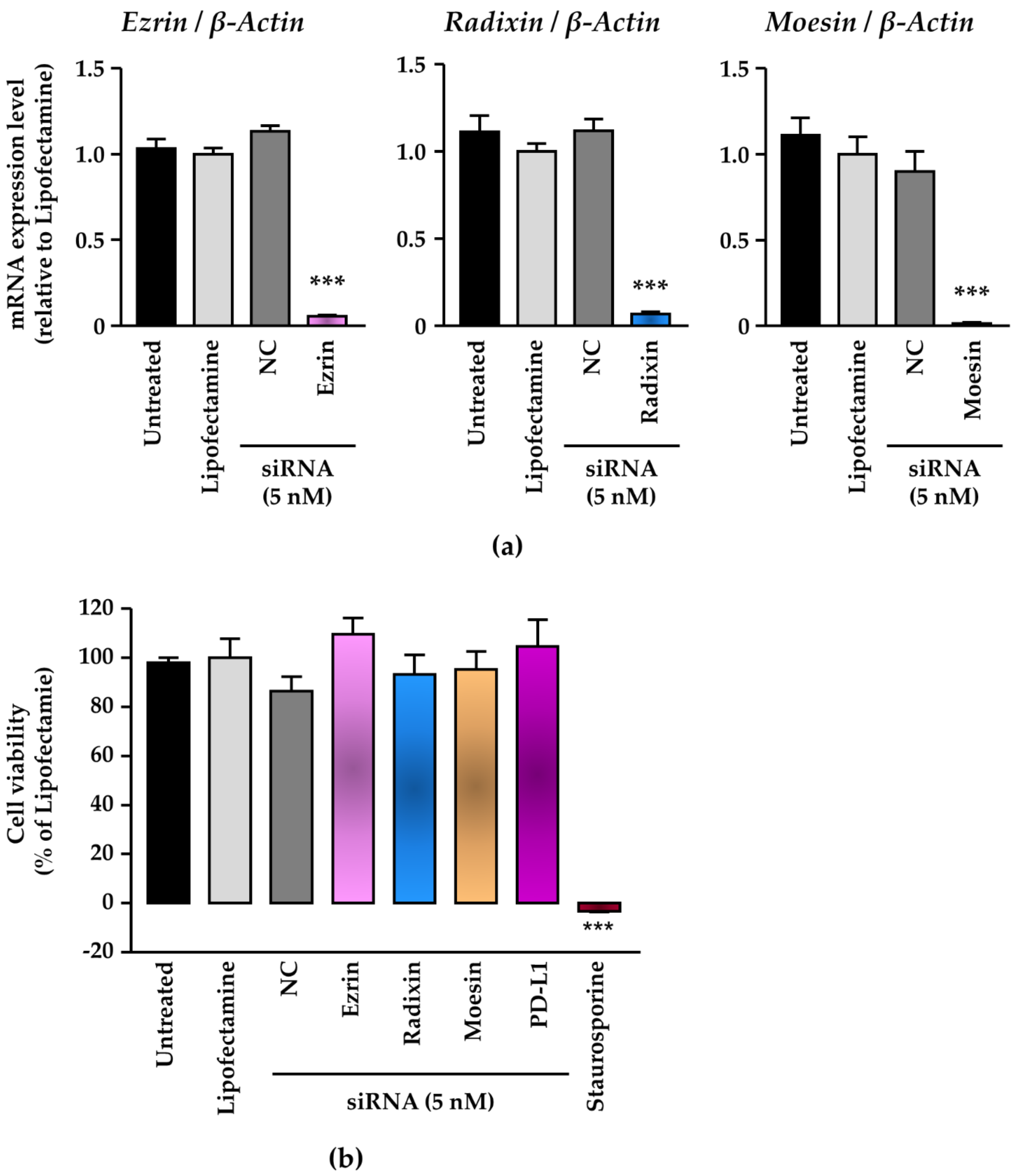

Figure S6. Effects of RNA interference-mediated knockdown of the ERM family on target mRNA levels in and cell viability of HCS-2 cells. Cells were cultured with transfection medium (Untreated), transfection reagent (Lipofectamine), nontargeting control (NC) siRNA, or the siRNAs against the target genes at 5 nM for three days. (**a**) mRNA levels of ezrin, radixin, and moesin normalized to that of β-actin and relative to that of Lipofectamine alone as determined by RT-PCR. $n = 3$, ***$p < 0.001$ vs. Lipofectamine. All data are expressed as the mean ± standard of the mean (SEM) and were analyzed by one-way analysis of variance (ANOVA) followed by Dunnett's tests. (**b**) Cell viability of HCS-2 cells. $n = 6$, ***$p < 0.001$ vs. Lipofectamine. All data are expressed as the mean ± SEM and were analyzed by one-way ANOVA followed by Dunnett's tests.

Moesin is a scaffold protein responsible for the cell surface expression of PD-L1 in HCS-2 cells

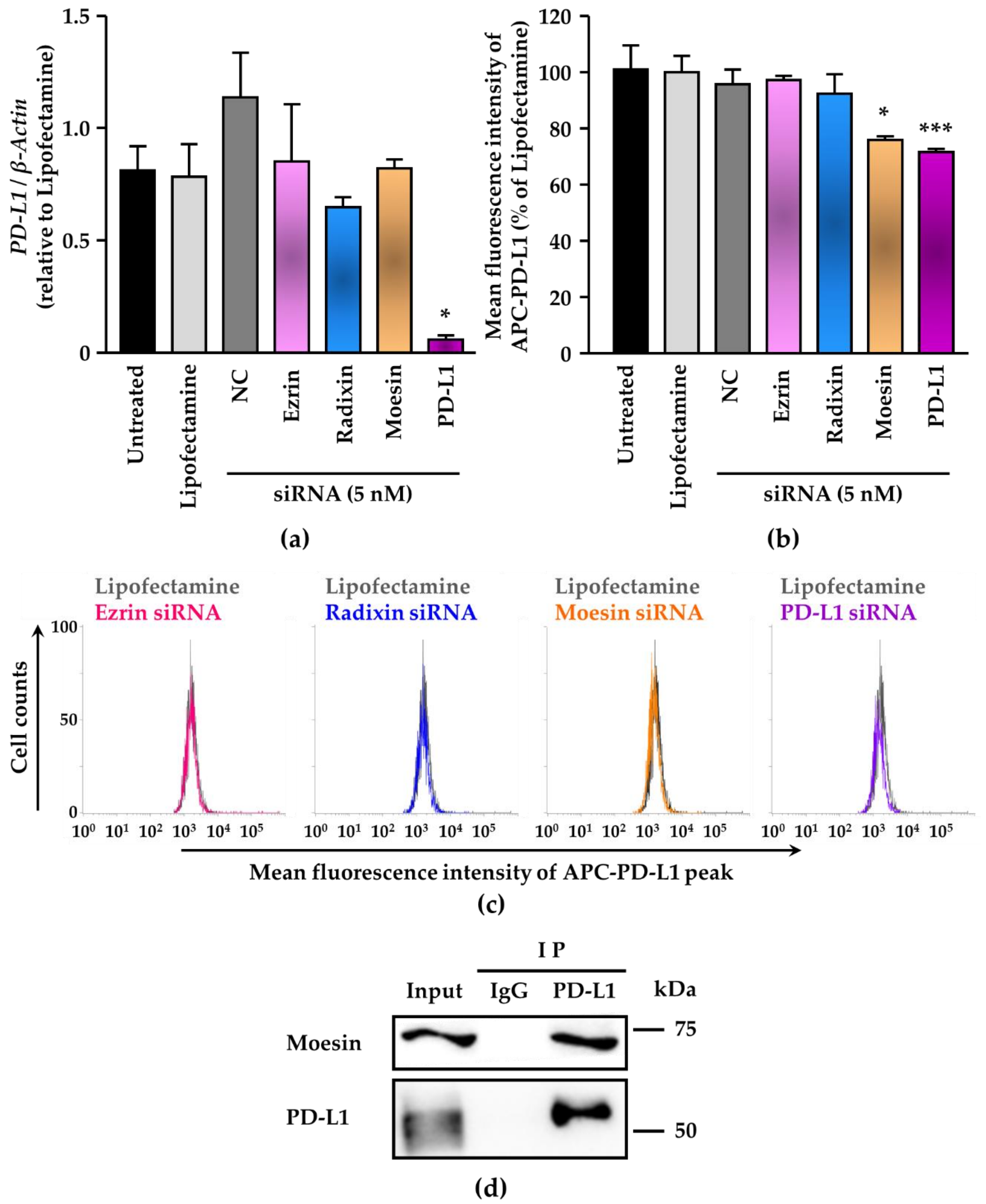

Figure S7. Moesin is a scaffold protein responsible for the cell surface expression of PD-L1 in HCS-2 cells. Cells were cultured with transfection medium (Untreated), transfection reagent (Lipofectamine), NC siRNA, or the siRNAs against the target genes at 5 nM for three days (**a**–**c**). (**a**) PD-L1 mRNA expression levels normalized to that of β-actin in cells relative to that in Lipofectamine-treated cells as determined by RT-PCR; $n = 3$, * $p < 0.05$ vs. Lipofectamine. (**b**) Mean fluorescence intensity of allophycocyanin (APC)-labeled PD-L1 in the surface plasma membrane relative to that in Lipofectamine treatment, quantified by flow cytometry; $n = 3$, *** $p < 0.001$, * $p < 0.05$ vs. Lipofectamine. All data are expressed the mean ± SEM and were analyzed by one-way ANOVA followed by Dunnett's tests. (**c**) Overlay of representative histograms of the mean fluorescence intensity of APC-PD-L1 in the surface plasma membrane of HCS-2 cells as measured by flow cytometry. (**d**) Representative immunoblots of moesin and PD-L1 in whole-cell lysates (input) and immune precipitates pulled down with a control IgG or an anti-PD-L1 Ab. Molecular weight is expressed in kDa.

Molecular interaction of PD-L1 with ezrin and radixin in BOKU cells

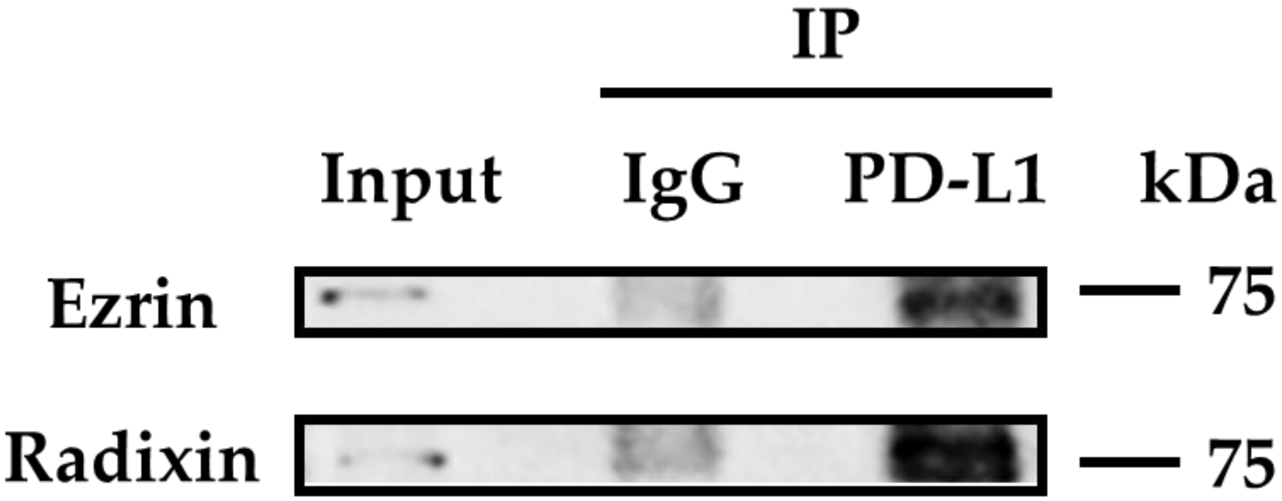

Figure S8. Molecular interaction of PD-L1 with ezrin and radixin in BOKU cells. Representative immunoblots of ezrin and radixin in whole-cell lysates (input) and immune precipitates pulled down by a control IgG or an anti-PD-L1 antibody. Molecular weight is expressed in kDa.

Table S1. List of primers used for real-time reverse transcription polymerase chain reaction

Gene	Primer sequence (5′→3′)
h-β-Actin (forward)	TGGCACCCAGCACAATGAA
h-β-Actin (reverse)	CTAAGTCATAGTCCGCCTAGAAGCA
h-Ezrin (forward)	ACCATGGATGCAGAGCTGGAG
h-Ezrin (reverse)	CATAGTGGAGGCCAAAGTACCACA
h-Radixin (forward)	GAATTTGCCATTCAGCCCAATA
h-Radixin (reverse)	GCCATGTAGAATAACCTTTGCTGTC
h-Moesin (forward)	CCGAATCCAAGCCGTGTGTA
h-Moesin (reverse)	GGCAAACTCCAGCTCTGCATC
h-PD-L1 (forward)	CAATGTGACCAGCACACTGAGAA
h-PD-L1 (reverse)	GGCATAATAAGATGGCTCCCAGAA

Table S2. List of antibodies used in this study

Antibodies	Source	Cat. No.	Dilution
rabbit anti-ezrin	Cell Signaling Technology	3145	1:2,000 (WB)<br>1:50 (IF)
rabbit anti-radixin	Gene Tex	GTX105408	1:2,000 (WB)<br>1:50 (IF)
rabbit anti-moesin	Cell Signaling Technology	3150	1:2,000 (WB)<br>1:50 (IF)
rabbit anti-moesin (phospho T558)	Abcam	Ab177943	1:50 (IF)
Alexa Fluor 488-conjugated rabbit anti-PD-L1	Cell Signaling Technology	25048	1:50 (IF)
Alexa Fluor 488-conjugated goat anti-rabbit IgG	Thermo Fisher Scientific	R37116	1:25 (IF)
Alexa Fluor 594-conjugated goat anti-rabbit IgG	Thermo Fisher Scientific	R37117	1:25 (IF)
HRP-conjugated rabbit anti-PD-L1	Cell Signaling Technology	51296s	1:1,000 (WB)
mouse anti-GAPDH	Merck	MAB374	1:20,000 (WB)
HRP-conjugated anti-rabbit IgG (heavy + light)	SeraCare Life Sciences	5220-0336	1:10,000 (WB)
HRP-conjugated anti-mouse IgG (heavy + light)	SeraCare Life Sciences	5220-0341	1:10,000 (WB)
rabbit anti-PD-L1	Cell Signaling Technology	13684	1:30 (IP)
rabbit IgG isotype control	Cell Signaling Technology	3900	1:30 (IP)
APC-conjugated mouse anti-human PD-L1	BioLegend	329708	2.8 μg/test<br>(FC in BOKU cells)<br>3.2 μg/test<br>(FC in HCS-2 cells)

western blotting; WB, immunofluorescence; IF, immunoprecipitation; IP, flow cytometry; FC, horseradish peroxidase; HRP, allophycocyanin; APC
